# Gender Differences in the Longitudinal Association between Work-Related Injury and Depression

**DOI:** 10.3390/ijerph13111077

**Published:** 2016-11-02

**Authors:** Jaeyoung Kim, Yeongchull Choi

**Affiliations:** Department of Preventive Medicine, College of Medicine, Keimyung University, Daegu 42601, Korea; yeongchull@gmail.com

**Keywords:** gender, depression, occupational injury, longitudinal study, bidirectional association

## Abstract

Little is known about gender differences in the association between occupational injury and depression. We investigated the bidirectional association and gender differences between work-related injury and depression using the same cohort in the US Medical Expenditure Panel Survey (MEPS). In Analysis 1, the association of occupational injury and subsequent depression was investigated from 35,155 employees without depression. Analysis 2 included 32,355 participants without previous injury and examined the association of depression and work-related injury. The multivariable-adjusted odds ratio was estimated using a discrete time-proportional odds model. Male workers who had experienced workplace injury were more vulnerable to post-injury depression than non-injured male workers (OR = 2.35, 95% CI: 1.52, 3.65). Female workers with depression were more prone to get injured at the workplace than the non-depressed female workers (OR = 1.44, 95% CI: 1.07, 1.96). These results did not hold in the reverse direction for both genders. Workers compensation benefit was positively associated with the risk of post-injury depression among males, whereas anti-depressant medication and duration of depression were related to workplace injury among females. Gender differences in the direction and associated factors of the relationship between occupational injury and depression highlight the need for gender-specific intervention to the vicious cycle of workplace injury and depression.

## 1. Introduction

It has been reported that persons with severe traumatic injuries are more vulnerable to become depressed than the rest of the population [1,2,3,4]. The reverse situation has also been demonstrated: individuals with pre-existing mental health problems or depressive symptoms show an increased likelihood of physical disability, or traumatic injury [5,6,7,8]. One study documented a bidirectional relationship between unintentional injury and depression, such that individuals who are exposed to more traumatic injuries are at increased risk of developing depression, and more severe depression is associated with a higher likelihood of subsequent traumatic injuries [9].

Depression and injury are common health problems in the working population [10,11]. Suggestive evidence exists for each direction of the association between occupational injury and depression; workers with occupational injury have been reported to be more likely to be depressed [12,13,14,15,16], and workers with depressive symptoms or psychosocial job stress have exhibited an increased likelihood of injury at work [17,18,19]. However, the empirical literature is limited by a lack of longitudinal studies in examining a reciprocal link between injury and depression in the workplace setting. In assessing the association between occupational injury and depression, methodological concerns regarding confounding and reverse causation are important because the positive findings may not be attributable to depression or injury per se, but may instead be created spuriously from confounding or reverse causation. Most previous cross-sectional studies have failed to address these concerns adequately. Only one study examined the bidirectionality of the relationship between traumatic injuries and depression in a prospective setting, but it was neither about workplace injury nor differentiated the effect by gender [9]. 

Previous studies often neglect gender specificity by adjusting for or matching gender in their analysis [20]. It has been shown that women have higher prevalence of depression than men [10,21], while men are more vulnerable to injury than are women [22,23]. However, no study has yet investigated simultaneously how the bidirectional association between occupational injury and depression differs by gender in a prospective setting. Therefore, the present study aimed to assess the bidirectionality and gender differences in the longitudinal association between work-related injuries and depression within the same cohort. Each direction of the association between occupational injury and depression was contrasted and the pattern of association by gender was examined for differences, using data from a nationally representative survey.

## 2. Materials and Methods

### 2.1. Study Population

The data source of this study was the 2000–2006 Medical Expenditure Panel Survey (MEPS), a nationally representative household survey on the US population [24]. The Agency for Healthcare Research and Quality (AHRQ) conducts a survey of households, individuals, and their health providers and employers using a multistage clustered sample design. The household component of the survey consists of reporting the cost and use of specific health services, and information related to the participants’ physical and mental health conditions. Health insurance coverage and job information are collected through insurance/employer components. Data are collected through 5 rounds of each panel at 4- to 5-month intervals, over two and a half years. A new panel of sample household is selected each year and data for each panel are collected for two calendar years through a series of 5 rounds of in-person interviews. The overall response rate across panels has generally ranged from 65 to 71 percent, while individual round response rates, subject to participation in the prior round of survey, are over 90 percent [25].

The longitudinal panel was constructed using the household respondents’ files for each year, which was then merged with the files on medical conditions and job information for each year. This study pooled six constructed MEPS panels from Panel 5 to Panel 10. We chose Round 2 as the baseline and used the information from Round 1 as an indicator for previous history of occupational injury, depression, and other comorbidity. Persons who had a previous history of injury or depression, as reported in Round 1 that was conducted 4–5 months earlier, were excluded from the analysis (*n* = 4955). Among the initial eligible total (IET) of 95,594 respondents from 6 pooled panels, persons were excluded if they: (1) did not complete the two-year survey in each panel due to death, departure from the US, institutionalization, or military service (*n* = 2573; 2.7% of IET); (2) were not eligible for all 5 rounds (*n* = 854) or were a proxy interview; (3) were aged less than 18 years or over 65 years (*n* = 38,410); (4) were unemployed at baseline (*n* = 12,857). The final study sample comprised 40,900 respondents.

### 2.2. Measures

Household Components survey respondents were asked to self-report on their health problems in the last 4–5 months, including physical conditions, accidents, injuries, and mental or emotional health conditions. Information on each condition reported by the Household Component respondent was recorded verbatim, and later coded by professional coders into appropriate International Classification of Disease, 9th Revision (ICD-9) codes. Reports of injury were retrieved from the medical condition files if respondents stated that “the medical condition they experienced during the four or five months since the previous interview” was “due to an accident or injury”. If the injury happened while the person was at work, it was identified as an occupational injury in that specific round. Only the first injury in each round was selected in order to differentiate continuing treatment of an injury condition from the previous round. The ICD-9 codes for injury were used to categorize the injured body region and type of injury based on the Barell classification matrix [26]. Injury severity was calculated using the Abbreviated Injury Scale (AIS) [27] on the ICD-9 codes.

Depression was identified using two ICD-9 codes: 296.2 (major depression, single episode) and 311 (depressive disorder, Not Elsewhere Classified (NEC)). For each report of depression in a specific round, corresponding information regarding the health care services that individuals used, such as prescribed anti-depressants, hospital inpatient services, ambulatory services, and visits to the emergency room (ER) was collected. The analysis was confined to only the first occurrence of a depression episode for each respondent across the five rounds. Using information on the number of prescribed medications, hospital inpatient services, ambulatory services, and ER visits, the measures of anti-depressant use, duration of depression, and number of depression episodes were constructed.

Most covariates were measured at baseline except some of the demographic variables. The following covariates were considered as potential confounders in the analysis, based on reported risk factors of depression and workplace injury in the literature [28,29,30]: demographic variables (age, gender, race, education, marital status, and family income level), job-related characteristics (occupational group, company size, self-employment, job tenure, and overtime work), medical condition (comorbidity, functional activity limitation, cognitive function impairment, self-rated physical and mental health, and number of health care events per condition), health behavior (current smoking status, alcohol or substance abuse problem, exercise, obesity), and access to health care (insurance coverage and having a particular doctor or health center that the individual visits regularly). To take comorbidity into account, we calculated a comorbidity index based on D’Hoore’s implementation of the Charlson comorbidity score [31]. Family income level was defined as the percentage of federal poverty line (FPL) (high: ≥400% FPL, middle: 200%–399% FPL, low: <200% FPL). Physical activity was measured based on whether the person engaged in moderate or vigorous physical activity 3 times per week. Current smoking was defined as having smoked at least 100 cigarettes in their lifetime and currently smokes every day or some days. Functional activity limitation was defined as having any activity limitation at work or home due to a medical condition. Cognitive function impairment included experience of confusion or memory loss, having problems making decisions, or requiring supervision for their own safety. Information on workers’ compensation (WC) benefit was assessed in terms of the total amount of payment received from “person’s workers’ compensation” per year. Injury severity was categorized based on the AIS score and Injury Severity Score (ISS). Based on the ISS, injury severity was rates as follows: ISS 1–8 = minor, ISS 9–15 = moderate, and ISS 16 or over = severe. Type of injury was categorized as musculoskeletal injury vs. others based on the ICD codes of the injury. Injury treatment duration was counted as the number of rounds the same injury episode was reported in a single panel. Time of depression occurrence after injury was indicated by the length of time taken for the occurrence of depression after the injury was reported. It was calculated by the number of rounds following the report of the first injury episode.

### 2.3. Analyses

Because the two objectives of our study required different population samples, here we described the analysis samples and procedures separately. To compare the associations in both directions, we estimated each direction separately rather than simultaneously, because doing so allows for more flexibility in specifying the model. Because the MEPS does not have information on the exact time when an event occurs, but only its interview round is identified, the data was treated as a grouped survival data and observations were tied to the interview. A discrete time-proportional odds model was chosen given that 5 of the tied observations in the data considered time as a discrete variable. The multivariable-adjusted ORs was estimated using a discrete time-proportional odds model [32]. Analyses were performed separately by gender. 

#### 2.3.1. Occupational Injury and Risk of Subsequent Depression (Analysis 1)

Analysis 1 included 35,155 persons without depression at baseline, and estimated the odds ratio (OR) of depression incidence at follow-up time for those with and without injury. We first compared the distribution of baseline characteristics by occupational injury using *t*-tests for continuous variables and χ^2^ tests for categorical variables.

For Analysis 1, which examined the impact of occupational injury on subsequent depression, we excluded persons with a previous history of depression (*n* = 1433) and those currently depressed at baseline (*n* = 548) from the final sample of 40,900. This was done to preserve the temporal relationship between exposure and outcome, as well as to reduce the possibility that depression would affect the likelihood of injury in the following rounds (reverse causation). We also excluded respondents who had reported injuries in any of the previous rounds (*n* = 3522) to avoid residual confounding from the presence of injury-prone characteristics at baseline. Lastly, respondents who had missing information related to the covariates (*n* = 242) were excluded. This left 35,155 persons as the final analytic sample. 

To explore the mechanisms explaining the relationship between injury and depression, we used a stepwise approach to get insight into the change in the OR from one step to another. If there was a practically important or clinically meaningful relationship between the variable and injury/depression, it was considered a potential confounder. A change in the estimated measure of association of 10% or more also was considered for assessing confounders. Age, race, and income were retained for the final model based on prior studies showing their associations with depression and injury. The final model for Analysis 1 included the following covariates: age; race; family income; health care accessibility; marital status; smoking; obesity; comorbidity; cognitive function impairment; occupational group; work status; and any activity limitation at work, house, or school due to medical conditions. Interactions between the main predictor and the relevant covariates were examined for inclusion in the final model. No statistically significant interaction was observed. The subgroup analyses by injury characteristics were conducted with restriction to a subset of workers with injuries.

#### 2.3.2. Depression and Risk of Occupational Injury (Analysis 2)

Analysis 2 included 32,355 persons without injury at baseline and calculated the OR of injury occurrence at the follow-up round for those with and without depression. For Analysis 2, to examine the impact of depression on occupational injury occurrence, persons were excluded if they had a previous history of injury (*n* = 3522) and injury at baseline (*n* = 2701), if they had a previous history of depression (*n* = 1433), and if they did not complete the self-administered questionnaire (SAQ) (*n* = 504), or had missing data on key covariates (*n* = 385). This left 32,355 persons for the final analysis. We compared the distribution of baseline characteristics by depression status using *t*-tests for continuous variables and χ^2^ tests for categorical variables. Then again we used a series of multivariate regression models to examine the relationship between depression and occupational injury occurrence. The modeling approach was the same as that described above. The subgroup analyses by depression characteristics were conducted with restriction to a subset of workers with depression.

All analyses were performed using SAS 9.3 (SAS Institute Inc., Cary, NC, USA). A statistically significant association between an exposure and the outcome was declared when the *p*-value was less than 0.05.

This study was exempt from the requirement for subject consent under category 4 (research of existing data publicly available) by the institutional review boards of Harvard School of Public Health.

## 3. Results

### 3.1. Occupational Injury and Depression

Table 1 summarizes the characteristics of the study population who did not have depression by baseline injury status. For both genders, compared with workers without injury, those with an occupational injury had lower levels of education, a lower level of family income, and a marital status of divorced, widowed, or separated. Additionally, for both genders, more injured workers tended to have no health care accessibility, more smoke currently and reported a poor grade of self-rated physical health. More male and female workers with an occupational injury worked in a blue-collar occupation. More female workers who were injured on the job tended to be obese, and to have activity limitation with comorbidity and worked in service occupations. Work characteristics such as job tenure or overtime work showed that male injured workers have slightly shorter job tenure and more often do overtime work than non-injured workers. Female non-injured workers were more often working as part time workers than were injured female workers.

In Analysis 1, after excluding the respondents with a previous history of injury and depression, as well as those with concurrent depression, the incidence of depression among males was 4.4% and 1.9% for those with and without an occupational injury, respectively. The corresponding figures for female workers were 7.8% and 4.5%, respectively. Table 2 shows the proportional odds of developing depression after an occupational injury by multivariate analysis. In the multivariate analysis, male workers showed positive associations between occupational injury and depression (OR, 2.35; 95% CI: 1.52, 3.65). Female workers also showed a similar pattern of elevated odds for depression after an occupational injury, but the association was no longer statistically significant following adjustment for covariates (OR, 1.31, 95% CI: 0.83, 2.06). For both gender, divorce, current smoking, obesity, activity limitation, and co-morbidity health were significantly associated with post-injury depression.

Subgroup analysis by WC benefit or injury characteristics were presented at Table 3. Among males, receiving WC benefits for medical payments was associated with 2.83 times (95% CI: 1.04, 5.49) higher odds of developing depression, but did not hold true for females. Those with more severe injuries and shorter duration of treatment showed greater odds of depression for both genders. For both genders, musculoskeletal injury was significantly associated with higher odds of developing depression than was any other type of injury. 

### 3.2. Depression and Occupational Injury

The characteristics of the study population who did not have a prevalent occupational injury at baseline by depression status have been summarized in Table 4. Findings revealed a similar distribution in terms of family income and occupational group between workers with and without depression, for both genders. Depressed workers, irrespective of gender, appeared to be more socioeconomically disadvantaged, with a lower level of education, and no health care accessibility. They also tended to be less likely to be married and more likely to currently smoke, to be obese, to rate their physical and mental health status as poor, and to have comorbidities, active limitations and impaired cognitive function. Female depressed workers were less physically active than were non-depressed female workers. For both genders, a greater proportion of workers with depression was engaged in part-time work and had been employed for less than one year. In Analysis 2, among males, the incidence of occupational injuries was 7.6% and 5.5% for depressed and non-depressed workers, respectively. Among female workers, the same was 5.1% and 3.5%, respectively.

Multivariate analyses (Table 5) showed that the relative odds of getting injured on the job were 1.24 times higher in male workers with depression than those without depression, though it was not statistically significant (95% CI: 0.97, 1.87). Among female workers, depression was associated with an increase in the odds of occupational injury (OR = 1.44, 95% CI: 1.07, 1.96). For female workers, current smoking, obesity, occupational group of service or blue collar were positively associated with odds of occupational injury.

Subgroup analysis by depression characteristics were presented at Table 6. Taking anti-depressant medication, a longer duration of depression, and a higher number of episodes of treatment for depression were associated with higher odds of occurrence of occupational injury among depressed female workers, but not among depressed males.

## 4. Discussion

This study analyzed whether there is a bidirectional association between occupational injury and depression within the same cohort of the US working population. Our findings suggest that a modest association does exist, but the association differs by gender. Male workers showed significantly higher risk of getting depressed after occupational injury, but female workers did not. Female workers with depression faced a greater risk of being injured on the job as compared to those without depression, but this association was not significant among male workers, as revealed in the multivariate model.

The directional strength of the association between depression and occupational injury was opposite in men and women. In other words, injured male workers show higher odds of getting depressed than non-injured male workers, while depressed female workers show higher odds of getting injured at the workplace than non-depressed female workers. The only study reporting a bidirectional relationship between occupational injuries and depression suggested a stronger effect in women [9]. However, it was not directly comparable to our study, because it combined the data on males and females and then compared the effect of depression/injury on both genders. Gender differences in the association between workplace injury and depression appear to be very complex. It appears to be a product of both differences in depression between male and female workers as well as in injury characteristics. Men and women are exposed to different working conditions and they react differently. The different levels of severity or nature of injury may also explain the differential impact of occupational injury and depression by gender.

Several studies have reported an association between occupational injury and depression in one direction or the other [9,18,29,33]. Risk factors for post-injury depression in our study were consistent with the previous studies: female gender, injury severity, and higher levels of perceived stress or pain [14,34,35]. Although female workers tend to get depressed more after an injury, our subgroup analysis by gender revealed a relatively higher impact of occupational injury on depression in males. A previous study from that used linked WC and medical insurance data found that the before- and after-injury increase was larger for men although fewer men were treated for post-injury depression [36]. This may suggest that men are more deeply affected by occupational injuries than are women. Men had greater sensitivity than women to the depressogenic effects of financial, occupational, and legal stress [37,38].

Our finding suggests that the psychological stress associated with the WC process may lead to elevated depression among male workers. We hypothesized that the distress related to litigation may act as an intermediate factor in the pathway from occupational injury to depression for male workers. A few studies have suggested that the very process of dealing with WC has a deleterious effect on the post-injury trajectory of injured workers [39,40]. In a Canadian study, the experience of litigation substantially explained the level of depression in workers who had suffered mild-to-moderate traumatic brain injury [41]. WC payments was a crucial factor in explaining male workers’ depression after an occupational injury, but this did not hold true for female workers. For both genders, more severe injury, musculoskeletal injury and longer time after the injury were significant predictors for post-injury depression. 

Our finding that depression was associated with higher risk of occupational injury occurrence among females is consistent with the previous study [17]. Peele reported that depression may serve as a precursor to occupational injury for women. In our study, taking anti-depressant medications yield significantly higher odds (OR = 1.43, 95% CI: 1.16, 1.75) for occupational injury among female workers. Like anti-depressants, increasing duration of depression and number of depression episodes showed a similar pattern of higher risks for occupational injury among females, but not among males. 

Most previous studies examining the relationship between anti-depressants and risk of injury have been confined to falls or fractures in the elderly [5,6,42], or traffic injuries in drivers [43]. Only few studies reported the psychotropic effects of medication and the risk of injury on the job, but their results were mixed [29]. In a case-control study conducted on the British population [18], anti-depressants were found to be related to a higher risk of occupational injury, and there were only marginal differences between gender in terms of this risk. A Canadian study using a national health survey [9] reported no association between anti-depressants and injury risk. The relatively higher impact of antidepressants on workplace injury risk in women implies that anti-depressants may play a different role in the association of depression and occupational injury between genders. Women seem to be more vulnerable to the detrimental effects of anti-depressants, especially in terms of occupational injury risk. This might be explained, at least partially, by different pharmacokinetics [44,45] and different occupational risk factors among men and women [46], including the fact that women more often occupy part-time jobs and that they potentially face different levels of exposure to danger and stress on the job.

Several limitations of the present study need to be considered. First, the dataset did not include information on several potential confounding variables, including family history, other stressful life events outside the workplace, detailed job descriptions, and psychosocial conditions in the workplace. Lack of this data limited the exploration of the potential mechanisms underlying the observed gender difference in the association between occupational injury and depression, and vice versa. Second, the information from the MEPS was self-reported. The self-reported depression/injury measure, if under-reported, was more likely to attenuate the observed association between occupational injury and depression. In sensitivity analysis of excluding participants with the self-reported mental health status of being “poor”, the association between occupational injury and depression was attenuated in both directions, regardless of gender (Supplementary Materials Table S1). Self-reported measures may also introduce recall bias, although the intervals between the rounds in this study were relatively short. Additionally, it was verified that the error rate for coding medical conditions based on the ICD-9 codes did not exceed 2.5% [47]. Therefore, it is unlikely that our results were biased by the self-reported data on medical conditions on the MEPS. Third, history of injury or depression was assessed based on data from Round 1. There was an interval of 4–5 months between subsequent interview rounds. A longer time period may have been necessary, especially for a history of depression to be evident. Additionally, due to these intervals, we were not able to capture outcomes soon after the onset of their injury/depression. Forth, there were not enough cases for male injury after depression for Analysis 2, which may lead to inadequate power to detect a relationship should one actually exist. The interpretation of results should be cautious accordingly. Finally, the attrition rate of the MEPS may not be random. The initial response rate on the MEPS was over 85%, but 30% of the respondents had been lost by the fifth round. This could lead to a potential bias, although the AHRQ reported that it had no evidence of potential non-response bias attributable to survey attrition on resultant national estimates of health care cost [48]. 

Despite these limitations, this study adds to the evidence on gender difference and directionality in the association between occupational injury and depression, within the same cohort, using a nationally representative population-based survey. By using the longitudinal feature of the MEPS—its repeated measures of occupational injury and depression over time—we were able to establish the temporality of the association between occupational injury and depression, and vice versa. The rich information for all medically treated or related health conditions enabled us to take into account comorbidity, activity limitation, and severity of injury or depression, which can affect the association between workplace injury and depression. In addition, by adjusting for potential confounders that may mask the true effect of depression or injury, and by excluding cases with pre-existing injury and depression at baseline, we were able to take into account the potential bias due to reverse causation.

## 5. Conclusions

Although women were more prone to be depressed in general, the relative effect of occupational injury on depression was larger on men. Despite the fact that men were more prone to get injured on the job, the relative impact of depression on occupational injury was higher for women. Our findings suggest that male workers who had experienced workplace injury are more vulnerable to post-injury depression than are non-injured male workers, and female workers with depression are more vulnerable to get injured at the workplace as compared to non-depressed female workers. Indeed, occupational injury and depression affect each other; however, its bidirectional relationship was different by gender. Our findings highlight the need to interrupt this vicious cycle of workplace injury and depression by implementing gender-specific interventions.

## Figures and Tables

**Table 1:** Characteristics of men and women without prevalent depression by occupational injury status in the Medical Expenditure Panel Survey (MEPS), 2000–2006.

	Male			Female		
Selected Characteristics ^†^	No Injury (*n* = 17,645)	Occupational Injury (*n* = 598)	*p*-Value	No Injury (*n* = 16,606)	Occupational Injury (*n* = 306)	*p*-Value
Mean age (SD), years	39.4(11.8)	39.0(11.3)	0.860	39.1(11.9)	40.6(11.5)	0.012
Race			0.049			0.564
White	12,752(72.3)	446(74.6)		11,149(67.1)	198(64.7)	
Black	1846(10.5)	44(7.4)		2529(15.2)	53(17.3)	
Other	3047(17.3)	108(18.1)		2928(17.6)	55(18.0)	
Education			<0.0001			0.007
Less than high school	4492(25.5)	179(29.9)		3289(19.8)	76(24.8)	
High school graduate	7811(44.3)	302(50.5)		7991(48.1)	153(50.0)	
College or more	4164(23.6)	74(12.4)		3955(23.8)	49(16.0)	
Other degree	1178(6.7)	43(7.2)		1371(8.3)	28(9.2)	
Marital status			0.012			<0.0001
Married	11,228(63.6)	383(64.1)		9174(55.2)	134(43.8)	
Never married	4678(26.5)	137(22.9)		4462(26.9)	90(29.4)	
Divorced, widowed, separated	1739(9.9)	78(13.0)		2970(17.9)	82(26.8)	
Family income			0.001			0.001
High	7080(40.1)	193(32.3)		6230(37.5)	80(26.2)	
Middle	5948(33.7)	230(38.5)		5476(33.0)	113(36.9)	
Low	4617(26.2)	175(29.2)		4900(29.5)	113(36.9)	
No health care accessibility	6253(35.7)	196(33.0)	0.176	3756(22.8)	51(16.7)	0.011
Health insurance coverage			0.109			0.076
Any private	13,051(74.0)	422(70.6)		12,654(76.2)	221(72.2)	
Public only	643(3.6)	29(4.8)		1304(7.8)	25(8.2)	
Uninsured	3951(22.4)	147(24.6)		2648(16.0)	60(19.6)	
No physical activity	6981(39.6)	236(39.5)	0.961	7662(46.1)	151(49.3)	0.264
Current smoking	3874(22.0)	174(29.1)	<0.0001	2906(17.5)	77(25.2)	0.001
Alcohol or substance abuse problem	28(0.2)	-	N/A	14(0.1)	1(0.3)	0.459
Obese (BMI ≥ 30)	4509(25.6)	170(28.4)	0.113	4341(26.1)	122(39.9)	<0.0001
Activity limitation	199(1.1)	9(1.5)	0.393	251(1.5)	13(4.3)	0.001
Cognitive function limitation	115(0.7)	5(0.8)	0.378	165(1.0)	2(0.7)	0.806
Co-morbidity ^∏^	1641(9.3)	49(8.19)	0.648	1457(8.8)	38(12.4)	0.130
Self-rated physical health: Poor	147(0.8)	10(1.7)	0.028	156(0.9)	14(4.6)	<0.0001
Self-rated mental health: Poor	32(0.2)	2(0.3)	0.393	42(0.3)	2(0.7)	0.172
Occupational group			<0.0001			0.001
White collar	7654(43.4)	150(25.1)		10,940(65.9)	168(54.9)	
Service	2188(12.4)	69(11.5)		3773(22.7)	95(31.1)	
Farm	271(1.5)	18(3.0)		84(0.5)	3(0.9)	
Blue collar	7127(40.4)	350(58.5)		1655(9.9)	38(12.4)	
Job tenure			0.002			0.378
Less than 1 year	4606(29.3)	218(30.2)		5110(34.1)	146(34.8)	
More than 5 years	7107(45.3)	310(43.0)		5771(38.5)	176(41.9)	
Overtime work	5293(31.4)	469(34.2)	0.038	2615(16.2)	143(18.8)	0.083
Work status: part time	3641(21.6)	310(22.6)	0.449	5989(37.1)	237(31.1)	0.004
WC payment	-	218(36.5)	N/A	-	131(42.8)	N/A
Incident cases of depression, No.	334(1.9)	26(4.4)	<0.0001	747(4.5)	24(7.8)	0.005
Person-round	12,421	1880		89,951	1735	

^†^ In some categories, numbers may not sum to total due to missing information. ^∏^ Charlson co-morbidity index ≥1.

**Table 2:** Association between occupational injury and subsequent depression in the MEPS population.

	Male		Female	
Selected Characteristics	OR ^a^	95% CI	OR ^a^	95% CI
Depression				
No occupational injury	1.00		1.00	
Occupational injury	2.35	1.52, 3.65	1.31	0.83, 2.06
Age	1.00	0.99, 1.01	0.98	0.99, 1.00
Race				
White	1.00		1.00	
Black	0.73	0.49, 1.07	0.55	0.43, 0.69
Other	0.92	0.69, 1.22	0.66	0.53, 0.83
Marital status				
Married	1.00		1.00	
Never married	1.11	0.82, 1.50	0.92	0.75, 1.14
Divorced, widowed, separated	1.78	1.32, 2.39	1.33	1.09, 1.60
Family income				
High	1.00		1.00	
Middle	1.26	0.98, 1.62	1.06	0.87, 1.29
Low	1.09	0.81, 1.48	1.57	1.28, 1.94
Health care accessibility				
Yes	1.00		1.00	
No	1.12	0.88, 1.43	1.43	1.18, 1.74
Current smoking				
No	1.00		1.00	
Yes	1.76	1.40, 2.21	1.71	1.45, 2.02
Obese (BMI ≥ 30)				
No	1.00		1.00	
Yes	1.38	1.09, 1.73	1.29	1.10, 1.52
Activity limitation				
No	1.00		1.00	
Yes	1.93	1.02, 3.65	2.28	1.55, 3.35
Co-morbidity				
Charlson co-morbidity index <1	1.00		1.00	
Charlson co-morbidity index ≥1	1.62	1.18, 2.22	1.61	1.29, 2.00
Occupational group				
White collar	1.00		1.00	
Service	0.72	0.31, 1.66	0.97	0.81, 1.16
Farm	0.47	0.14, 1.50	0.19	0.02, 1.43
Blue collar	0.90	0.70, 1.14	0.83	0.64, 1.08
Work status				
Full time	1.00		1.00	
Part time	1.22	0.94, 1.58	1.11	0.95, 1.30

^a^: model was adjusted for age, race, education, family income, health care accessibility, marital status, smoking, obesity, co morbidity, occupational group, work status, and any activity limitation at work, house, or school due to medical condition.

**Table 3:** Proportional odds of depression by injury characteristics among occupationally injured MEPS population, stratified by gender.

	Male		Female	
	OR ^a^	95% CI	OR ^a^	95% CI
Workers’ compensation				
No	1.00		1.00	
Yes	2.83	1.04, 5.49	0.90	0.35, 2.24
Injury severity (ISS)				
Minor (<9)	1.00		1.00	
Moderate (9~15)	1.74	0.86, 3.59	0.89	0.35, 1.81
Severe (≥16)	2.89	1.68, 4.69	1.80	1.03, 3.12
Type of injury				
all other	1.00		1.00	
Musculoskeletal	2.16	1.09, 4.19	1.86	1.05, 3.25

^a^: model was adjusted for age, race, education, family income, health care accessibility, marital status, smoking, obesity, co morbidity, occupational group, work status, and any activity limitation at work, house, or school due to medical condition.

**Table 4:** Characteristics of men and women without prevalent injury by depression status in the MEPS, 2000–2006.

	Male			Female		
Selected Characteristics ^†^	No Depression (*n* = 16,426)	Depression (*n* = 159)	*p*-Value	No Depression (*n* = 15,434)	Depression (*n* = 336)	*p*-Value
Mean age(years)	39.4(11.8)	41.2(12.5)	0.065	39.1(11.9)	40.9(11.8)	0.005
Race			0.009			0.001
White	11,885(72.4)	132(83.0)		10,365(67.2)	255(75.9)	
Black	1700(10.3)	8(5.0)		2342(15.2)	31(9.2)	
Other	2841(17.3)	19(12.0)		2727(17.7)	50(14.9)	
Education			0.003			0.014
Less than high school	3984(24.2)	36(22.6)		3783(24.5)	71(21.1)	
High school graduate	3376(20.6)	48(30.2)		3897(25.2)	109(32.4)	
Some college	4831(29.4)	50(31.5)		4689(30.4)	87(26.0)	
College graduation or more	4235(25.8)	25(15.7)		3065(19.9)	69(20.5)	
Marital status			<0.0001			<0.0001
Married	10,647(64.8)	82(51.5)		8684(56.3)	161(47.9)	
Never married	4164(25.4)	45(28.3)		3978(25.7)	72(21.4)	
Divorces, widowed, separated	1615(9.8)	32(20.1)		2772(18.0)	103(30.7)	
Family income			0.714			0.183
High	6574(40.0)	62(39.0)		5777(37.4)	117(34.8)	
Middle	7925(48.3)	75(47.2)		7354(47.7)	157(46.7)	
Low	1927(11.7)	22(13.8)		2303(14.9)	62(18.5)	
No health care accessibility	5864(35.7)	53(33.3)	0.016	3534(22.9)	66(19.7)	<0.0001
Health insurance coverage			0.770			0.004
Any private	12,101(73.7)	119(74.8)		11,737(76.1)	254(75.6)	
Public only	581(3.5)	5(3.2)		1178(7.6)	40(11.9)	
Uninsured	3744(22.8)	35(22.0)		2519(16.3)	42(12.5)	
No physical activity	6560(39.9)	69(43.4)	0.375	7120(16.1)	180(53.6)	0.006
Current smoking	3627(22.1)	58(36.5)	<0.0001	2691(17.4)	93(27.7)	<0.0001
Alcohol or substance abuse problem	23(0.1)	2(1.3)	0.0003	25(0.2)	4(1.2)	<0.0001
Obese (BMI ≥ 30)	4238(25.8)	52(32.7)	0.049	4105(26.6)	123(36.6)	<0.0001
Activity limitation	182(1.1)	9(5.7)	<0.0001	227(1.5)	19(5.6)	<0.0001
Cognitive function limitation	145(0.9)	7(4.4)	<0.0001	128(0.9)	35(4.0)	<0.0001
Co-morbidity ^∏^	511(3.1)	34(7.1)	<0.0001	540(3.5)	19(5.8)	0.001
Self-rated physical health: Poor	137(0.8)	2(1.3)	0.559	145(0.9)	14(4.2)	<0.0001
Self-rated mental health: Poor	28(0.2)	4(2.5)	<0.0001	36(0.2)	10(3.0)	<0.0001
Occupational group			0.558			0.877
White collar	7096(43.2)	77(48.4)		10,174(65.9)	213(63.4)	
Service	1989(12.1)	17(10.7)		3472(22.5)	82(24.4)	
Farm	264(1.6)	1(0.6)		79(0.5)	2(0.6)	
Blue collar	6712(40.9)	62(39.0)		1569(10.2)	35(10.4)	
Job tenure			0.048			0.020
Less than 1 year	5404(32.9)	72(45.3)		5263(34.1)	140(41.7)	
More than 5 years	7423(45.2)	55(34.6)		5952(38.5)	119(35.4)	
Work status: part time	3412(21.0)	52(32.7)	0.0002	556(36.1)	146(43.5)	0.005
Incident cases of occupational injury	904(5.5)	12(7.6)	0.261	543(3.5)	17(5.1)	0.131
Person-round	80,359	760		46,931	1193	

^†^ In some categories, Numbers may not sum to total due to missing information. ^∏^ Charlson co-morbidity index ≥1.

**Table 5:** Association between occupational injury and subsequent depression in the MEPS population.

	Male		Female	
Selected Characteristics	OR ^a^	95% CI	OR ^a^	95% CI
Occupational injury				
No depression	1.00		1.00	
Depression	1.24	0.97, 1.87	1.44	1.07, 1.96
Age	0.99	0.99, 1.00	1.01	1.00, 1.02
Race				
White	1.00		1.00	
Black	0.72	0.56, 0.91	0.92	0.72, 1.17
Other	0.91	0.75, 1.09	1.03	0.82, 1.30
Marital status				
Married	1.00		1.00	
Never married	1.16	0.96, 1.40	1.14	0.9, 1.44
Divorced, widowed, separated	1.49	1.21, 1.82	1.07	0.85, 1.34
Family income				
High	1.00		1.00	
Middle	1.26	1.07, 1.47	1.17	0.95, 1.44
Low	0.93	0.72, 1.19	1.51	0.85, 1.54
Health care accessibility				
Yes	1.00		1.00	
No	1.17	1.01, 1.36	1.26	1.01, 1.57
Current smoking				
No	1.00		1.00	
Yes	1.48	1.28, 1.72	1.54	1.27, 1.88
Obese (BMI ≥ 30)				
No	1.00		1.00	
Yes	1.08	0.93, 1.26	1.45	1.21, 1.75
Activity limitation				
No	1.00		1.00	
Yes	1.17	0.68, 2.03	1.65	0.99, 2.75
Co-morbidity				
Charlson co-morbidity index <1	1.00		1.00	
Charlson co-morbidity index ≥1	1.14	0.90, 1.44	0.99	0.75, 1.32
Occupational group				
White collar	1.00		1.00	
Service	1.77	1.39, 2.25	1.52	1.24, 1.87
Farm	2.61	1.61, 4.22	1.31	0.40, 4.22
Blue collar	2.56	2.16, 3.03	1.38	1.06, 1.81
Work status				
Full time	1.00		1.00	
Part time	1.03	0.87, 1.22	0.77	0.63, 0.93

^a^: model was adjusted for age, race, education, family income, health care accessibility, marital status, smoking, obesity, co morbidity, occupational group, work status, and any activity limitation at work, house, or school due to medical condition.

**Table 6:** Proportional odds of occupational injury by depression characteristics among depressed workers in the MEPS population, stratified by gender.

	Male		Female	
	OR ^a^	95% CI	OR ^a^	95% CI
Anti-depressant medication				
No	1.00		1.00	
Yes	1.08	0.67, 1.75	1.43	1.16, 1.75
Duration of depression				
1 round	1.00		1.00	
More than 1 round	1.27	0.89, 1.84	1.42	1.07, 1.87
Number of depression episodes				
One	1.00		1.00	
More than one	1.09	0.65, 1.82	1.53	1.07, 2.17

^a^: model was adjusted for age, race, education, family income, health care accessibility, marital status, smoking, obesity, co morbidity, occupational group, work status, and any activity limitation at work, house, or school due to medical condition.

## Supplementary Materials

The following are available online at www.mdpi.com/1660-4601/13/11/1077/s1, Table S1: Association between occupational injury and depression in the MEPS population after excluding participants with low mental health status.
