# Supplementary material for: Gender Differences in the Longitudinal Association between Work-Related Injury and Depression
Source: Int J Environ Res Public Health. 2016 Nov 2;13(11):1077. doi: 10.3390/ijerph13111077 (PMC5129287; doi:10.3390/ijerph13111077)
Supplement: Supplementary file 1 [file ijerph-13-01077-s001.pdf]

Supplementary Materials: Gender Differences in the Longitudinal Association between Work-Related Injury and Depression

Jaeyoung Kim and Yeongchull Choi

Table S1. Association between occupational injury and depression in the MEPS population after excluding participants with low mental health status.

	Male		Female	
	**OR** [a]	**95% CI**	**OR** [a]	**95% CI**
Odds of depression				
No occupational injury	1.00		1.00	
Occupational injury	2.26	1.49, 3.42	1.55	1.00, 2.38
Odds of occupational injury				
No depression	1.00		1.00	
depression	1.17	0.62, 2.19	1.29	0.78, 2.14

[a]: model was adjusted for age, race, education, family income, health care accessibility, marital status, smoking, obesity, co morbidity, occupational group, work status, and any activity limitation at work, house, or school due to medical condition.

© 2016 by the authors; licensee MDPI, Basel, Switzerland. This article is an open access article distributed under the terms and conditions of the Creative Commons by Attribution (CC-BY) license (http://creativecommons.org/licenses/by/4.0/).
